# Investigation on the Evolution of Shiga Toxin-Converting Phages Based on Whole Genome Sequencing

**DOI:** 10.3389/fmicb.2020.01472

**Published:** 2020-07-10

**Authors:** Michele Zuppi, Rosangela Tozzoli, Paola Chiani, Pablo Quiros, Adan Martinez-Velazquez, Valeria Michelacci, Maite Muniesa, Stefano Morabito

**Affiliations:** ^1^Department of Food Safety, Nutrition and Veterinary Public Health, Istituto Superiore di Sanità, Rome, Italy; ^2^Department of Genetics, Microbiology and Statistics, University of Barcelona, Barcelona, Spain

**Keywords:** Shiga toxin, phages, Stx2, ecology, evolution, WGS

## Abstract

Bacteriophages are pivotal elements in the dissemination of virulence genes. The main virulence determinants of Shiga Toxin producing *E. coli*, Shiga Toxins (Stx), are encoded by genes localized in the genome of lambdoid bacteriophages. Stx comprise two antigenically different types, Stx1 and Stx2, further divided into subtypes. Among these, certain Stx2 subtypes appear to be more commonly occurring in the most severe forms of the STEC disease, haemorrhagic colitis and haemolytic uremic syndrome (HUS). This study aimed at obtaining insights on the evolution of Stx2 bacteriophages, due to their relevance in public health, and we report here on the analysis of the genomic structure of Stx2 converting phages in relation with the known reservoir of the *E. coli* strains harboring them. Stx2-converting phages conveying the genes encoding different *stx2* subtypes have been isolated from STEC strains and their whole genomes have been sequenced, analyzed and compared to those of other Stx2 phages available in the public domain. The phages’ regions containing the *stx2* genes have been analyzed in depth allowing to make inference on the possible mechanisms of selection and maintenance of certain Stx2 phages in the reservoir. The “*stx* regions” of different *stx2* gene subtypes grouped into three different evolutionary lines in the comparative analysis, reflecting the frequency with which these subtypes are found in different animal niches, suggesting that the colonization of specific reservoir by STEC strains could be influenced by the Stx phage that they carry. Noteworthy, we could identify the presence of *nanS-p* gene exclusively in the “*stx* regions” of the phages identified in STEC strains commonly found in cattle. As a matter of fact, this gene encodes an esterase capable of metabolizing sialic acids produced by submaxillary glands of bovines and present in great quantities in their gastrointestinal tract.

## Introduction

Shiga Toxin-producing *E. coli* (STEC) are pathogens of public health concern, capable of inducing severe disease in humans, either at the gastrointestinal or systemic level (Caprioli et al., 2005). STEC disease includes different symptoms, ranging from non-complicated diarrhea to haemorrhagic colitis and the Haemolytic Uremic Syndrome (HUS), which represents the most severe form of STEC infection.

The STEC pathotype consists in a heterogeneous group of intestinal pathogenic *E. coli* and owes the name to the ability of elaborating one or more toxins known as Shiga toxins (Stx), whose activity can block the protein synthesis in target eukaryotic cells, causing their death (Melton-Celsa, 2014). Stx-coding genes are carried by lambdoid prophages, referred to as Stx-converting phages or Stx phages (O’Brien et al., 1983; Huang et al., 1987; Bryan et al., 2015) and encode two main antigenically distinct toxin types, Stx1 and Stx2. Each of these types is further divided into subtypes, three for Stx1 (Stx1a, Stx1c and Stx1d) and at least seven for Stx2 (Stx2a, Stx2b, Stx2c, Stx2d, Stx2e, Stx2f and Stx2g) (Scheutz et al., 2012), although new Stx2 subtypes are continuously described, including Stx2h to k (Lacher et al., 2016; Bai et al., 2018; Yang et al., 2020). The Stx and their subtypes have been associated with different frequency to the onset of severe diseases (Fuller et al., 2011; Melton-Celsa, 2014; Krüger and Lucchesi, 2015). In fact, Stx2 is more frequently associated, compared to Stx1, to HUS and haemorrhagic colitis and even more striking is the association between HUS and the subtypes Stx2a, Stx2c and Stx2d (Fuller et al., 2011; Melton-Celsa, 2014; Krüger and Lucchesi, 2015).

STEC are zoonotic pathogens and, although cattle have been recognized as the main asymptomatic animal reservoir (Karmali, 2018), these bacteria can be found in the intestinal tract of many animals, including other ruminants, pigs and pigeons (Mughini-Gras et al., 2018). It is important to note that STEC strains producing different Stx2 subtypes can be associated with different animal reservoirs. The *E. coli* strains producing Stx2a, Stx2c and to a lesser extent Stx2d, are mainly isolated from the bovine reservoir (Januszkiewicz and Rastawicki, 2016; Akiyama et al., 2017; Jajarmi et al., 2017; Tostes et al., 2017; Cha et al., 2018; Rahman et al., 2018; Dehdashti et al., 2019). Other subtypes, such as the Stx2b and Stx2g, can be also found in STEC isolated from ruminants but with a lower prevalence (Januszkiewicz and Rastawicki, 2016; Akiyama et al., 2017; Jajarmi et al., 2017; Dehdashti et al., 2019). The Stx2e subtype is produced by STEC found almost exclusively in pigs (Krüger et al., 2011; Cha et al., 2018), while strains producing the Stx2f subtype seem to preferentially or exclusively colonize pigeons (Krüger and Lucchesi, 2015; Van Hoek et al., 2019). An explanation for such observations has not been proposed yet. In general, it is conceivable that STEC strains producing distinctive Stx2 subtypes may have acquired advantages facilitating the survival in the specific reservoir.

The role of prophages is crucial in the microbial evolution (Javadi et al., 2017), as they may increase the evolutionary fitness of their host by favoring the rearrangement of genomic regions and the acquisition of foreign DNA providing new features through Horizontal Gene Transfer (Feiner et al., 2015). In particular, the spreading of Stx phages contributed to the emergence of different Shiga toxin-producing *E. coli* types (e.g., Stx-producing Enteroaggregative *E. coli* (Navarro-Garcia, 2014) and Stx producing Enterotoxigenic *E. coli* (Michelacci et al., 2018), among others), by providing the ability of producing the Stx to different diarrheagenic *E. coli*.

In this study, we focused on the evolution of Stx2 phages conveying the genes encoding different Stx2 subtypes based on whole genome sequencing data. We isolated and sequenced a set of Stx2 phages and analyzed their genomes in comparison to a panel of Stx2 phages’ genomes available in the public domain in the attempt to shed light on their evolution and correlating this analysis with the Stx subtype conveyed.

## Materials and Methods

### Bacterial Strains

The STEC strains used for the induction of the phages were part of the culture collections of the European Union Reference Laboratory for *E. coli* and the University of Barcelona and are listed in Table 1. The *E. coli* K12 strain XL1-Blue (Bullock et al., 1987) was used as propagator strain for the amplification of the induced phages.

**TABLE 1:** Bacteriophages isolated and sequenced in this study together with sequence quality controls.

Phage	Stx subtype	*E. coli* strain source	*E. coli* Serotype	Number of sequencing reads obtained	Average PHRED quality
ΦEH250	Stx2b	EH250	O118:H12	57,620	30
Φ62	Stx2c	62 Stx2-r	O171:H2	45,531	31
Φ63	Stx2c	63 Stx2-r	O171:H2	42,872	32
Φ72	Stx2c	72 Stx2-r	O181:H20	47,616	30
Φ75	Stx2e	75 Stx2-r	O2:H21	67,044	32
Φ148	Stx2e	148 Stx Fr1.20	O100:H-	43,326	31
Φ86	Stx2g	86 Stx2-r	O2:H25	105,927	32
Φ89	Stx2g	89 Stx2-r	O2:H25	98,913	32
Φ93	Stx2g	93 Stx2-r	O2:H25	55,906	31

### Induction and Amplification of the Bacteriophages

The STEC strains were treated with Mitomycin C to promote the induction of the Stx bacteriophages. In detail, the bacterial strains were grown in Luria-Bertani (LB) broth overnight at 37°C, diluted 1:100 into 10 ml of LB broth supplemented with 5 mM CaCl_2_ and grown with vigorous shaking (200 rpm) at 37°C, until an OD_600_ of 0.3 was reached. The cultures were then added with Mitomycin C (final concentration 0.5 μg/ml) and further incubated overnight in the dark. The treated cultures were finally filtered through sterile 0.22 μm Millex-GP filters (Merck KGaA, Darmstadt, Germany) to prepare the phage suspensions for the following amplification and purification. One hundred microliters of the phage suspensions were added to 100 μl of culture of the propagator strain XL1-Blue, grown in LB broth to 0.3 OD_600_, and incubated in a thermostatic bath at 37°C for 20 min. The cultures were added to 3.5 ml of LB modified soft agar (LB broth with 0.001% thiamine V/V and 5 mM CaCl_2_ with agar 7 g/l), kept at 46°C, and instantly poured on LB agar plates, which were then incubated overnight at 37°C.

Five milliliters of SM buffer (5.8 g NaCl, 2 g MgSO_4_⋅7 H_2_O, 50 ml Tris-HCl 1 M pH 7.5, 0.1 g gelatin, 950 ml deionized H_2_O) were dispensed to each plate to recover the phage particles that underwent lytic cycle and kept at 4°C overnight. The phage suspensions in SM buffer were recovered, added with chloroform at 5% final concentration and centrifuged twice at 4500 × g at 4°C for 20 min, in order to recover the phage particles from the supernatant while eliminating bacterial and agar remains. The phage suspensions were then used to re-infect the propagator strain XL1-Blue, as described above, in order to increase the phage titre, recovered with SM buffer and concentrated with Amicon^®^ Ultra-15 Centrifugal Filter Units with Ultracel-30 tubes (Merck KGaA, Darmstadt, Germany) with a cut-off of 30 kDa.

### Purification in CsCl Gradient and Extraction of the Phage’s DNA

The phage suspensions were purified by CsCl density gradients, following the protocol described by Sambrook and Russel (2001). Briefly, 2 ml of sucrose 20% (w/v) was first added to the tubes to ultra-Clear BECKMAN^®^ tubes (Life Sciences Division Headquarters, Indianapolis, IN, United States) before adding 1 ml of CsCl solutions of density of 1.7, 1.5, and 1.3 g/ml, respectively. The different solutions of CsCl were sequentially added for the creation of the gradient. The phage suspensions were added with 0.5 g/ml of solid CsCl, incubated until the CsCl salts had completely dissolved and eventually 1 ml of the phage suspension containing CsCl was deposited above the gradient. The tubes containing the phages on the CsCl gradient were centrifuged at 80,000 × g for 2 h at 4°C in a Swinging-Bucket Rotor (Beckman, rotor SW41 Ti) (Life Sciences Division Headquarters, Indianapolis, IN, United States). The bands resulting from the centrifugation of the samples containing the phage particles were collected with a sterile syringe by puncturing the tube, added to a tube filled with a CsCl solution (density of 1.5 g/ml) and centrifuged again at 160,000 × g for 24 h at 4°C. Collection of the band was repeated, and the final suspension was dialyzed against 10 mM NaCl, 50 mM Tris-HCl pH 8.0, 10 mM MgCl_2_.

Each suspension was added with 100 units/ml of Sigma-Aldrich^®^ DNase I RNase-free (Merck KGaA, Darmstadt, Germany) and incubated at 37°C for 1 h for the removal of free DNA. The phages’ suspensions were treated with 50 μg/ml of Proteinase K Roche^®^ (Merck KGaA, Darmstadt, Germany) for 1 h at 56°C for the disruption of the phage capsid, and the DNA was extracted with phenol-chloroform-isoamyl alcohol, as reported by Sambrook and Russel (2001).

When more than one band was visualized, they were punctured separately and PCR targeting *stx* genes was carried out as described below to verify which band corresponded to the Stx phage.

### PCR Amplification and Typing of the Stx-Coding Genes

End-point PCR amplifications were performed with a GeneAmp PCR system 2400 (Perkin-Elmer, PE Applied Biosystem, Waltham, MA, United States). A 378 bp fragment of the *stx2*A subunit was amplified with primers UP378/LP378 (Muniesa and Jofre, 1998) that detected all *stx2* subtypes, except *stx2f*. The *stx* genes subtypes carried by the different phages were confirmed by PCR using the primers and amplification conditions described by Scheutz et al. (2012).

### Electron Microscopy Studies

Phage suspensions were 100-fold concentrated using Amicon Ultra 15 ml tubes (100 kDa Amicon Ultra centrifugal filter units, Millipore, Bedford, MA, United States) by centrifuging at 3,000 × g for 10 min. Ten microliter of each phage suspension were dropped onto copper grids with carbon coated Formvar films, negatively stained with 2% ammonium molybdate (pH 6.8) and examined under a Jeol 1010 transmission electron microscope (TEM) (JEOL Inc. Peabody, MA, United States) operating at 80 kV.

### Whole Genome Sequencing of the Phages DNA

The DNA libraries were prepared using the NEBNext^®^ Fast DNA Fragmentation & Library Prep Set for Ion Torrent (New England Biolabs, Ipswich, MA, United States). The template preparation and sequencing run were performed with an Ion One Touch 2 System and an Ion Torrent PGM sequencer (Life Technologies, Carlsbad, CA, United States) following the manufacturer’s instructions for 400 bp DNA libraries on an Ion 314^TM^ chip v2 (Life Technologies, Carlsbad, CA, United States).

### *In silico* Analysis of the Phage Genomes

The reads resulting from the sequencing of the phage genomes (Table 1) were uploaded to the public server ARIES^1^ for the bioinformatics analyses (Knijn et al., 2020). The quality of the reads was assessed using the tool FASTQC *(Galaxy v0.63)*^2^. The reads were then trimmed with “FASTQ positional and quality trimming” (Galaxy *v0.0.1*) (Cuccuru et al., 2014), in order to remove the segments corresponding to the Ion Torrent adapters and the regions presenting a Phred Quality Score for each base < 26. The reads were assembled through the software “SPAdes genome assembler for regular and single-cell projects” (Galaxy *v3.11.1*) (Bankevich et al., 2012). The contigs shorter than 1000 bp were filtered out [“Filter SPAdes output” (Galaxy *v0.1*)]. The filtered contigs were annotated with the tool “Prokka” (Galaxy *v1.12.0*) (Seemann, 2014), using both the bacterial and viral databases, together with the genomes recovered from the public domain (Table 2). The annotation of these latter genomes, already annotated, was repeated to avoid possible differences due to the use of different annotation tools.

**TABLE 2:** Bacteriophages genomes retrieved from GenBank.

Phage	*E. coli* serotype	Stx subtype	Acc. N°
WGPS9	O157:H7	Stx2a	AP012535.1
Min27	O157:H7	Stx2a	NC_010237.1
F403	O157:H7	Stx2a	AP012529.1
F422	O157:H7	Stx2a	AP012531.1
933W	O157:H7	Stx2a	AF125520.1
F451	O157:H7	Stx2a	AP012532.1
Sakai	O157:H7	Stx2a	NC_000902.1
PA45	O157:H7	Stx2a	KP682389.1
PA27	O157:H7	Stx2a	KP682380.1
PA5	O157:H7	Stx2a	KP682373.1
PA28	O157:H7	Stx2a	KP682381.1
F723	O157:H7	Stx2a	AP012533.1
TL-2011c	O103:H25	Stx2a	NC_019442.1
phiON-2011	O104:H4	Stx2a	KU298437.1
P13363	O104:H4	Stx2a	HG803182.1
P13803	O2:H27	Stx2a	HG792102.1
P8983	O104:H4	Stx2a	HG792103.1
P14437	O104:H4	Stx2a	HG792105.1
P13771	O104:H4	Stx2a	HG792104.1
phi191	O111:H2	Stx2a	NC_028660.1
phi272	O157:H7	Stx2a	NC_028656.1
PA8	O157:H7	Stx2a	KP682374.1
F765	O157:H7	Stx2a	AP012534.1
II	O157:H7	Stx2a	NC_004914.3
I	O157:H7	Stx2a	AP004402.1
86	O86:H-	Stx2a	NC_008464.1
P12009	O103:H25	Stx2a	AP010958.1
Xuzhou21	O157:H7	Stx2a	CP001925.1
2011c_3493	O104:H4	Stx2a	CP003289.1
2009EL_2050	O104:H4	Stx2a	CP003297.1
TW14359	O157:H7	Stx2c	CP001368.1
WGPS8	O157:H7	Stx2c	AP012540.1
WGPS4	O157:H7	Stx2c	AP012538.1
F349	O157:H7	Stx2c	AP012530.1
1717	O157:H7	Stx2c	NC_011357.1
2851	O157:H7	Stx2c	FM180578.1
WGPS6	O157:H7	Stx2c	AP012539.1
WGPS2	O157:H7	Stx2c	AP012537.1
1447	O157:H7	Stx2c	AP012536.1
2861	Ont:H20	Stx2d	NZ_LOIR01000058.1
2591	O174:H2	Stx2d	NZ_LOII01000068.1
2236	O113:H4	Stx2d	NZ_LOGY01000073.1
2595	O17/O77/O44:H18	Stx2d	NZ_LOIJ01000001.1
P27	Ont:H-	Stx2e	AJ298298.1
12_246M	O148:H18	Stx2e	QZVZ00000000.1
phi467	O26:H11	Stx2f	LN997803.1

The presence of the *stx* genes in all the genomes was assessed through the web application *“virulence finder”*^3^. The contigs that did not display the *stx* genes in the output were further manually screened using the NCBI BLAST+ blastn algorithm.

The annotated sequences were visualized with the software Snapgene^®^ (*v4.1.9*) (GSL, Biotech), through which it was possible to localize, isolate and characterize the gene composition of the “*stx* regions,” defined as the phage’s genome region encompassed by the genes *q* and *s* and containing the Stx-coding genes.

### Phylogenetic and Structural Analysis of the Phages’ Whole Genomes and “*stx* Regions”

All the genomes of the phages and the respective “*stx* regions” were compared through the software for multiple sequences alignment “MAFFT Multiple alignment program for amino acid or nucleotide sequences” (Galaxy Version 7.221.3) (Katoh and Standley, 2013). The alignment algorithm included the following parameters: L-INS-i, most accurate; recommended for < 200 sequences; with the iterative refinement method (max. 1000 iterations) incorporating local pairwise alignment information. We have used 0 as “Gap extend penalty” value, in order to eliminate the differences due to the alignment of sequences of different lengths. The alignments were used to produce a maximum likelihood tree with the ultrafast bootstrap based on 1000 replicates, visualized through the online software iTOL (v 4.3.2) (Letunic and Bork, 2016). The comparative analysis based on the structure of the phage genomes was carried out by computing the distance matrix based on the presence or absence of each ORF with an annotated function and using the GC content of each of the ORF as an additional criterion (Supplementary Table S1). In detail, each ORF with the same annotation was considered as being a different allele when displaying a different GC content. The presence, absence and the different allelic types were all used to compute the Hamming distance between pairs of samples by using the function “Distance matrix” (ver. 0.1.9) of the MentaLIST software (Feijao et al., 2018) (galaxy version 0.2.3). The matrix obtained was eventually used to produce a Neighbor Joining tree with the function “MentaLIST Tree” of the same software. The comparison of the phage structures was carried out using the BLAST algorithm embedded into the Circoletto online tool^4^ using the ultra-strict parameter (*E*-value-180) (Darzentas, 2010). For the pictogram construction, bit-score values have been used to describe the quality of the alignment at a given point, displayed in different colors (blue ≤ 50%; green ≤ 75%, orange ≤ 99%, and red = 100% identity). The bit-score is a normalized version of the score value returned by the BLAST searches, expressed in bits (Altschul et al., 1990).

## Results

### Stx Phage Isolation

**FIGURE 1:**
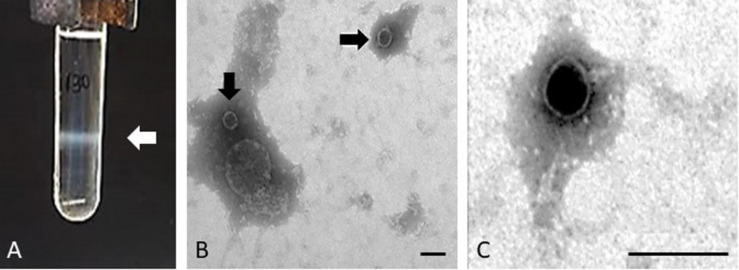
Phage purification. Example of the band comprising the Stx2 phage of isolate 62 and corresponding to a density of 1.5 g/ml in gradient of CsCl **(A)** and electron micrographs of Stx phage Φ62 **(B,C)**. Bar 100 nm.

The purification of the Stx phages from the majority of the STEC strains allowed the observation of one single band after the CsCl density gradients, indicative of the presence of one single prophage in enough density to be visualized (Figure 1A). The band was collected and the presence of the *stx* in phage DNA was confirmed by PCR (data not shown). Strains EH250 and 86 Stx2-r (Table 1) showed two bands which, after collection and DNA extraction, were tested by PCR to determine which of the two corresponded to Stx2b phage ΦEH250 and Stx2g phage Φ86, respectively. The second band in these strains plausibly corresponded to another non-Stx inducible phage present in the chromosome that was not used in this study.

Since some strains induce Stx prophages at low rates and in order to increase the volume of each Stx phage suspension to guarantee enough DNA for whole genome sequencing, several bands from different CsCl tubes were collected for each phage, purified and pooled together prior to DNA extraction. Some Stx phages were isolated in enough concentration to be visualized by electron microscopy. Phages ΦEH250, Φ62, Φ63, and Φ72 showed the same morphological type corresponding to phages of icosahedral capsids of 50 ± 3 nm of diameter with very small tails compatible with phages of the *Podoviridae*, a family of viruses in the order Caudovirales (King et al., 2011; Figures 1B,C).

### Whole Genome Sequencing of the *stx* Phages

The whole genome sequencing of the phages produced a number of reads ranging from 42,872 to 105,927 (Table 1). All the sequences produced were of good quality with an average PHRED value per base of 30–32 (Table 1). When the sequences were assembled, most of them yielded one or two contigs of total length > 39,000 bp, approximating the reported dimensions of the Stx phages’ genomes (Krüger and Lucchesi, 2015). The only exception was the genome of the phage ΦEH250, which was assembled into five different short contigs (Table 3). All the contigs produced were characterized by a high coverage values (145X-496X), with the exception of the contigs from the phage ΦEH250 sequence, whose coverage values ranged from 4.5X to 45.09X (Table 3).

**TABLE 3:** Characteristics of the assemblies of the phages’genomes sequenced in this study and identification of the presence of *stx2* genes.

Phage	Size of the contigs obtained in *bp* (Coverage)	*stx2* genes presence	GC content
ΦEH250	10 538 (4.5X); 11 870 (45.09X); 14 111 (38.2X); 16 478 (5.08X); 19 830 (6.57X)	Yes	50%
Φ62*	54 000 (171X)	Yes	50%
Φ63*	54 001 (145X)	Yes	50%
Φ72*	52 624 (148X)	Yes	51%
Φ75	39 345 (230X); 37 576 (77X)	No	48%
Φ148*	42 911 (178X)	Yes	49%
Φ86	39 942 (496X)	No	46%
Φ89	39 942 (408X)	No	47%
Φ93	39 942 (295X)	No	47%

*The whole genome sequences of the phages Φ62, Φ63, Φ72, and Φ148, produced in this study, were uploaded on the EMBL ENA database. (Study Acc. N°: PRJEB37181).

The *stx* gene subtypes carried by the different phages, identified by PCR on the DNA from the STEC strains or on the purified phage DNA (data not shown), were confirmed through the analysis of the draft genomes using the web application *“virulence finder”*^5^ for five out of the nine phages studied. The *stx* genes couldn’t be found in the sequenced genomes of the phages Φ75, Φ86, Φ89, and Φ93 (Table 3), either using the virulence finder application or by manually searching the contigs through the use of the NCBI BLAST+ blastn algorithm. This was probably caused by the sequencing of a phage, which did not possess the Stx2-coding genes that was present in the same CsCl band together with the one encoding the Stx. It is possible that the non-Stx phage was preferentially selected during the propagation of the phages. Interestingly, the phages that did not display the presence of the Stx-coding genes had on average a lower GC content of 46–48% with respect to the phages with the genes coding for the different Stx subtypes, which were all characterized by a GC content comprised between 49 and 51% (Table 3).

The whole genome sequences of the phages Φ62, Φ63, Φ72, and Φ148 were used for the following analyses and uploaded on the database EMBL ENA (Study Accession No. PRJEB37181, Table 3).

### Analysis of the “*stx* Regions”

**FIGURE 2:**
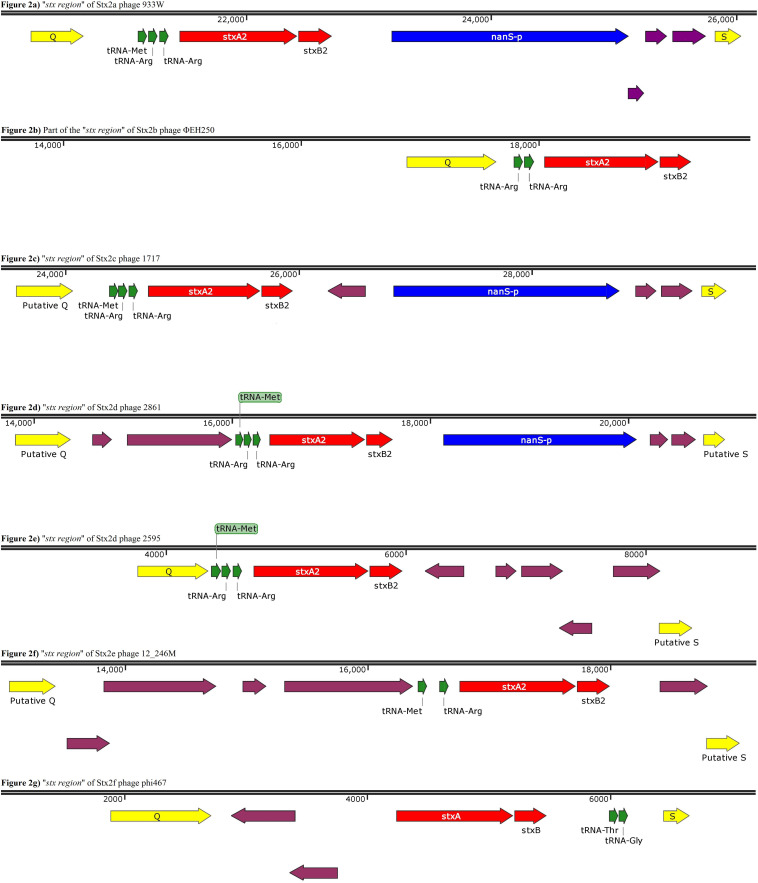
Panels **(a)** to **(g)** show the gene organization of gene organization of the “*stx* region” of Stx2 phages encoding different Stx2 subtypes. Panel **(b)** shows a contig displaying a partial “*stx* region” as from this phage an entire region was not obtained. The purple arrows indicate ORFs without an annotated function.

The regions of the draft phage genomes containing the Stx-coding genes, located between the phage genes *q* and *s* and named hereafter the “*stx* regions,” were identified and extracted from all the annotated genomes and visualized using the software Snapgene^®^ (*v4.1.9*) (GSL, Biotech). The genomic structure of these regions was analyzed in more detail (Figure 2). The “*stx* region” identified in the phage ΦEH250 was not complete, lacking the part comprised between the Stx-coding genes and the *s* gene, probably due to the problems encountered during the assembly of this phage’s genome (Figure 2B).

This analysis showed that the structure of the “*stx* regions” presented a common arrangement of features, which seemed to be associated with the subtype of Stx-coding genes conveyed. In particular, all the “*stx* regions” investigated presented a set of tRNA-coding genes that varied among the subtypes (Figure 2). These genes were always positioned between the late-phase regulator gene *q* and the *stx* genes, except for the Stx2f phage phi467, in which the “*stx* region” showed an opposite orientation regarding the *q* and *s* genes, when compared to the regions of the other phages analyzed, with the tRNA-coding genes placed close to the *s* gene (Figure 2G). In detail, the “*stx* region” of the phages encoding the Stx2a, Stx2c, and Stx2d all presented three genes for tRNA methionine, arginine and arginine (Figure 2), except for the phage TL-2011c (Table 2), encoding the Stx2a subtype, which presented the tRNA triplet methionine, proline and arginine (data not shown). The Stx2e phages all presented only two genes coding for the tRNA, encoding the tRNA methionine and arginine (Figure 2F), while the Stx2f phage also presented two of these genes, coding for the tRNA threonine and glycine (Figure 2G). Finally, the Stx2b phage analyzed presented two genes coding both for tRNA arginine (Figure 2B).

The “*stx* region” of all the Stx2a, Stx2c, and Stx2d phages differed from the regions of the phages encoding the other Stx2 subtypes also for the presence of the *nanS-p* gene (Figure 2), positioned downstream of the *stx* genes, with the exception of the Stx2d phage, 2595, which lacked this open reading frame (Figure 2E).

The gene *nanS-p* is the phage homolog of the bacterial gene *nanS*, which codes for an esterase involved in the metabolism of the 5-*N*-acetyl-9-*O*-acetyl neuraminic acid (Neu5,9Ac2) (Saile et al., 2018).

### Phylogenetic and Structural Analysis of Whole Genomes and “*stx* Regions” of the Stx2 Phages

The multiple sequence alignment analysis of the whole genomes and of the “*stx* regions” of the phages didn’t include the phages Φ75, Φ86, Φ89, and Φ93 as they didn’t show the presence of the *stx* genes (Table 3).

The phage ΦEH250 (Table 1) was excluded from the analysis as well. As matter of fact, the assembly of the draft genome of this phage did not yield contigs of sufficient length to be representative of the whole genome and the “*stx* region” was fragmented into different contigs.

**FIGURE 3:**
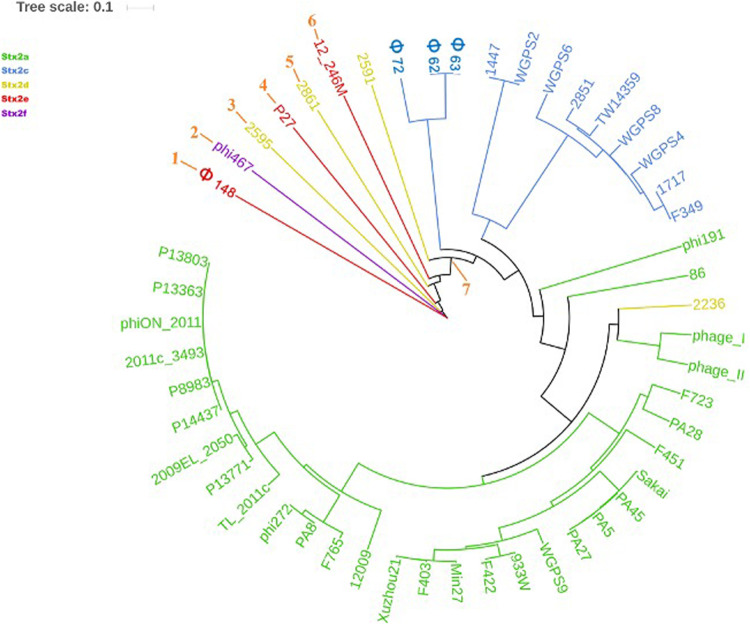
Dendrogram generated from the multiple sequence alignment of the whole genomes of the Stx2 phages with the MAFFT software. The different clades are numbered in orange at the node or the tip of the branch. The Maximum Likelihood tree has been built to display the topology with 95% bootstrap coverage estimated from 1000 repetitions and annotated using the iTOL software.

**FIGURE 4:**
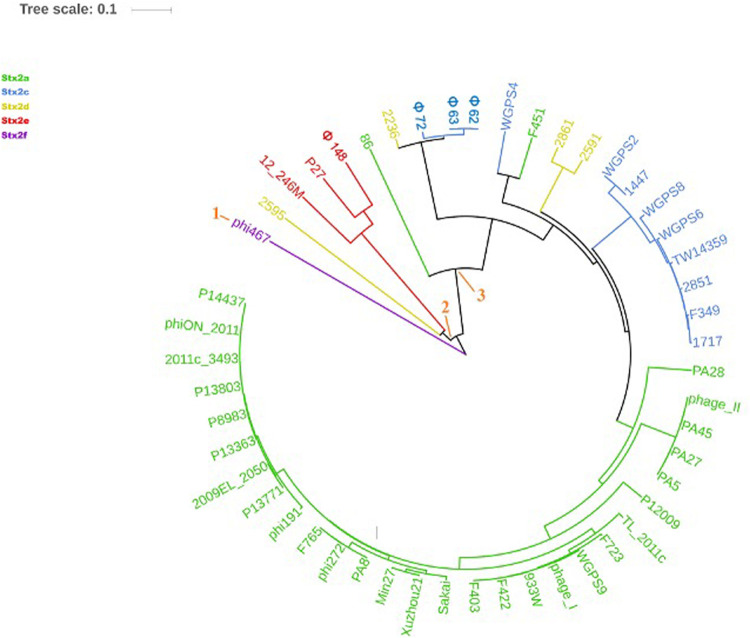
Dendrogram generated from the multiple sequence alignment of the “*stx* regions” of the Stx2 phages with the MAFFT software. The different clades are numbered in orange at the node or the tip of the branch. The Maximum Likelihood tree has been built to display the topology with 95% bootstrap coverage estimated from 1000 repetitions and annotated using the iTOL software.

**FIGURE 5:**
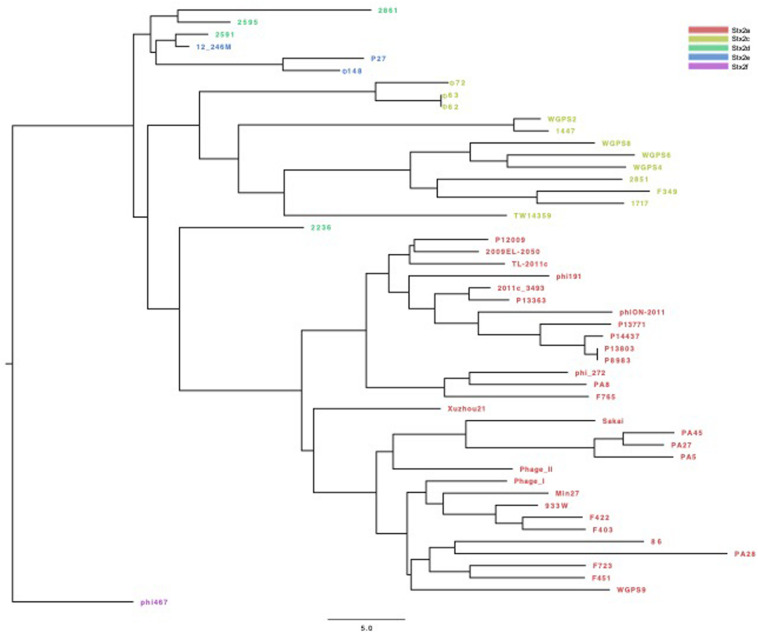
Dendrogram of relationships between the Stx phages’ genomes excluding the “*stx* regions.” The Neighbor Joining tree was constructed by comparing the presence and the GC content of the annotated ORFs in the different phages. Each ORF with the same annotation was considered as being a different allele when displaying a different GC content. The presence, absence and the different allelic types were all used to compute the Hamming distance. The phages are colored differently according to the Stx2 gene subtypes.

**FIGURE 6:**
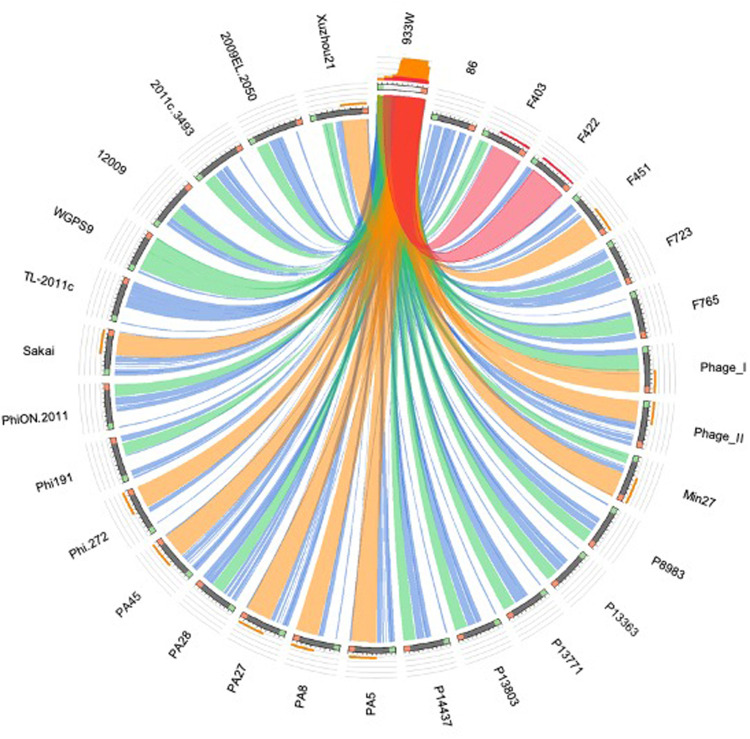
Sequence similarities between the s*tx*-phages carrying the genes encoding the Stx2a subtype. The picture shows the results of the BLAST local alignments using BP933W as a query against all the Stx2a-phage. The colors codes blue, green, orange and red represent the overall quality of the aligned segments along the phage sequences, evaluated on the basis of the bit-score values in the worst-to-the-best order (blue to red, blue ≤ 50%; green ≤ 75%, orange ≤ 99%, and red = 100% identity). The bit-score is a normalized version of the score value returned by the BLAST searches, expressed in bits. The height of the colored bars in the histogram on the top of the BP933W ideogram shows how many times each color hits a specific fragment of the other phage sequences. A twist in a ribbon indicates that the local alignment is inverted (query and database sequence on opposite strands).

**FIGURE 7:**
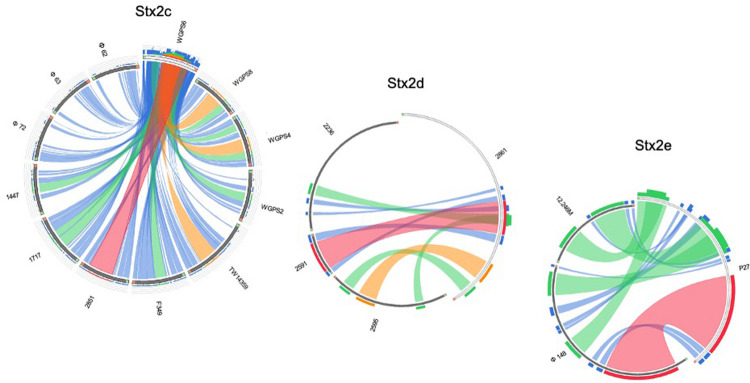
Sequence similarities between the s*tx*-phages carrying the genes encoding the Stx2c, Stx2d, and Stx2e subtypes. The picture shows the results of the BLAST local alignments using WGPS6, 2861, and P27, respectively, as queries against all the Stx2-phages belonging to the related Stx2 subtypes. The colors codes blue, green, orange and red represent the overall quality of the aligned segments along the phage sequences, evaluated on the basis of the bit-score values in the worst-to-the-best order (blue to red, blue ≤ 50%; green ≤ 75%, orange ≤ 99%, and red = 100% identity). The bit-score is a normalized version of the score value returned by the BLAST searches, expressed in bits. The height of the colored bars in the histogram on the top of the query ideograms shows how many times each color hits a specific fragment of the other phage sequences. A twist in a ribbon indicates that the local alignment is inverted (query and database sequence on opposite strands).

The phylogenetic analyses were conducted by comparing the whole genome sequences of the phages (Figure 3) and their “*stx* regions” (Figure 4). An additional analysis was carried out considering the presence and the absence of the annotated ORFs together with their GC content (Figure 5). All the dendrograms obtained analyzing the phage genomes showed that the Stx2a and Stx2c phages constituted homogenous populations descending from single ancestors. On the other hand, the Stx2d and Stx2e phages displayed a topology suggesting a relationship between these two populations with both the approaches (Figures 3, 5), although additional analyses of more sequence data would be needed to strengthen this result. Interestingly, both the dendrograms obtained with the whole genome sequences and the presence of the annotated ORFs produced the same topology (Figures 3, 5), with the phage 2236 (Stx2d) interspersed in the clade containing the phages encoding Stx2a and Stx2c, which seemed to originate from a single ancestor (Figures 3, 5). Noteworthy, all the phylogenetic analyses were concordant in placing the Stx2f phage into a single clade distant from all the other phages (Figures 3–5). The comparative analysis of the phage genomes confirmed the phylogenetic analysis and showed extensive variability within the same populations of phages encoding the different Stx2 subtypes, either in term of sequence homology or in the presence of phage’s regions (Figures 6, 7). Interestingly, the phage 2236, encoding Stx2d, was largely different from the other three phages carrying the genes coding for the same Stx2 subtype (Figure 7). This observation is in agreement with what observed with the phylogenetic analyses based either on the identification of differences in the phage sequences through multiple alignment (Figure 3) or on the presence of specific phage genes (Figure 5). This phage was also different from the other Stx2d phages and more similar to the Stx2a phages (Supplementary Figure S1).

The analysis of the “*stx* regions” carried out in the attempt to obtain information on the source of this DNA, which represents the transduced region of the phage’s genome, showed that the “*stx* regions” fell into three major clades (Figure 4), one composed, again, by the Stx2f phage phi467 only, one comprising the Stx2e phages and the Stx2d phage 2595, and the last clade including the Stx2a, Stx2c and the remaining Stx2d phages.

## Discussion

The bacteriophages encoding the Shiga toxins (Stx) are a heterogeneous group of lambdoid phages whose members can present different morphologies and variability in the host infectivity range (Krüger and Lucchesi, 2015).

While sharing the presence of the Shiga toxin genes and a few other characteristics, these phages show a considerable genomic diversity (Krüger and Lucchesi, 2015), which has not been thoroughly described, yet. Most of the studies present in the literature were performed with a limited number of phages (Recktenwald and Schmidt, 2002; Smith et al., 2012; Steyert et al., 2012; Tozzoli et al., 2014; Zhang et al., 2018), didn’t distinguish between the *stx* subtypes (Smith et al., 2007) or investigated the phage population present in STEC strains with respect to other features, such as the effect of stress factors on the induction of the prophages (Zhang et al., 2020).

In this study we meant to investigate the evolution of the phages encoding Stx2 belonging to different subtypes through whole genome sequencing. The choice to restrict this study to the phages encoding the Stx2 was due to the observation that this toxin type is associated with the most severe forms of the infections in humans (Krüger and Lucchesi, 2015). In order to do so, the genomes of four Stx2 phages, purified from STEC strains and sequenced in this study, were compared through multiple sequence alignment to the genomes of 46 other Stx2 phages found in NCBI GenBank. The same approach was used to investigate the structure of the phage regions conveying the Stx2-coding genes, the “*stx* region,” of all the bacteriophages analyzed.

Alongside other mobile genetic elements, phages are one of the most important engines of bacterial evolution, contributing enormously to their genetic variability through Horizontal Gene Transfer (Javadi et al., 2017). The phages genomes are known to be highly mosaic, being composed by segments with different evolutionary histories, which were acquired by phages through a variety of mechanisms, such as homologous recombination, transposition and site-specific recombination (Hatfull and Hendrix, 2011). Events of genomic rearrangement such as recombination involving regions of the host, as well as regions of other phages, active or cryptic, have been proposed to drive the evolution of the Stx phages, representing an important factor for their diversity (Smith et al., 2012).

The phylogenetic analysis of the whole genomes of the phages investigated in this study confirmed the significant diversity already described among the Stx phages (Zhang et al., 2020), even within the same toxin subtype (Figures 3, 5–7 and Supplementary Figure S1), suggesting that they could, in certain cases, have descended from different ancestors that acquired the Shiga toxin genes in independent transduction events. The phages encoding the Stx2a and Stx2c subtypes seemed to be part of a single lineage deriving from a common ancestor (Figures 3, 5). Interestingly, the same lineage seemed to have originated also at least one of the Stx2d phages, 2236, which shares a higher degree of homology in the sequence and gene content with the Stx2a phages than with those encoding the Stx2d subtype (Figures 3, 5 and Supplementary Figure S1). This observation may indicate that the Stx2d phages population may be the result of multiple events of transduction of the Stx genes operated by different phages. The remaining Stx2d phages seemed to be closer to those carrying the genes encoding the subtype Stx2e. STEC producing this latter Stx2 subtype are almost exclusively found in swine and the genomes of the Stx2e phages shared the same clade with the Stx2d phages 2861, 2595, and 2591 (Figures 3, 5). This finding is not surprising, since the Stx2d phages can be isolated from a variety of different niches besides bovines, including pigs (Baranzoni et al., 2016).

The Stx2f phages are found in *E. coli* strains whose natural reservoir corresponds to pigeons. These animals have a gastric physiology different from ruminants and it is conceivable that the *E. coli* part of the pigeon intestinal microbiota may have evolved differently from those found in ruminants. Accordingly, the phylogenetic and structural analyses showed the phage phi467, carrying the Stx2f-coding genes, falling into a clade separated from the others (Figures 3–5). We did not include many Stx2f-encoding phages in this study as they are not available in the public databases for downloading, but previous evidences showed that these phages share a very high degree of similarity (Grande et al., 2016). It is thus conceivable that they could have descended from a common ancestor and possibly remained confined to the pigeon host.

The “*stx* regions” of the phages investigated showed lower variability compared to the whole genomes and were divided into three clades (Figure 4). These overlapped only partially the topology of the dendrogram obtained through the whole genome analyses, reinforcing the hypothesis of the occurrence of multiple transduction events, with some of the “*stx* regions” carrying the same Stx2 subtype-coding genes present in phages belonging to different clades, as observed for the Stx2d and, to a lesser extent, Stx2e bacteriophages. This hypothesis may be also supported by the presence of the same tRNA genes in the *stx* region of phages carrying the same Stx2 subtype. In this respect, the tRNA gene sequences may be considered as a marker for the region captured by the ancestor phages during the excision from the bacterial chromosome and may have been involved in the transduction event.

Interestingly, the three clades observed in the phylogenetic analysis of the “*stx* regions” reflected, to a certain level, the frequency with which the Stx2 subtypes are encoded by *E. coli* strains isolated from the animal reservoirs. As a matter of fact, the “*stx* regions” of the Stx2a, Stx2c and most of the Stx2d phages were included into a single line (line 3, Figure 4). As reported previously, these are the subtypes which are commonly produced by *E. coli* strains isolated from ruminants, especially bovines.

It is known that prophages can improve the fitness of their bacterial host by carrying genes that can provide an evolutionary advantage, in a phenomenon known as “lysogenic conversion” (Feiner et al., 2015), of which the Shiga toxin could be an example. As a matter of fact, the Stx phages have been proposed to augment the fitness of certain *E. coli* K12 and clinical isolates of *E. coli* O157 by conferring protection against grazing protozoan (Steinberg and Levin, 2007). However, this observation has not been extended to other STEC serogroups and other authors have reported the lack of a protective effect provided by the Stx on protozoan predation of *E. coli* (Schmidt et al., 2016). As a matter of fact, at least in the laboratory conditions, they could not observe a differential decline of the STEC population with respect to other *E. coli*. Additionally, the same was observed when the grazing experiments were conducted using a wild type STEC and its lysogenic mutant, lacking the Stx phage (Schmidt et al., 2016). These observations make this aspect a gap yet to be filled in the knowledge of the evolution of the STEC strains and their Stx phages. In this respect, the *nanS-p* gene present in the “*stx* regions” of certain phages could be an example illustrating the lysogenic conversion. This gene has been identified in this study only in the phages conveying the genes encoding Stx2a, Stx2c and Stx2d subtypes, which are mainly present in *E. coli* strains isolated from ruminants. As reported previously, the *nanS-p* gene is a homologous of the gene *nanS*, located in the *E. coli* sialoregulon, which encodes an esterase able to mediate the release of 5-acetyl-neuroamminic acid (Neu5Ac) from Neu5,9Ac_2_ and from bovine submaxillary gland mucin (Saile et al., 2018). Neu5Ac, by disabling the repressor NanR, activates the *E. coli* sialoregulon, from which it is then utilized to produce pyruvate, used for the tricarboxylic acid cycle (Saile et al., 2018). Saile et al. (2018) suggested that the activity of additional NanS esterase could provide an evolutionary advantage to the bacteria by facilitating the utilization of sialic acids as an ATP source. Therefore, this gene could promote the colonization of the bovine reservoir through the degradation and utilization of bovine submaxillary mucin’s sialic acids.

Interestingly, the STEC strains that carry the phages conveying the subtypes Stx2a, Stx2c and Stx2d are also those most often linked to severe disease, during human infection (Koutsoumanis et al., 2020). Additional to the advantage offered to the *E. coli* in the ruminants’ gastro-intestinal (GI) tract, the *nanS-p* could also cause, as a side effect, an improved colonization of the human GI tract, rich of Neu-5,9-Ac_2_ (Saile et al., 2018), facilitating the insurgence of the most severe forms of the infections in humans. The only exception was represented by the Stx2d phage 2595, which did not show the presence of this gene and whose “*stx* region” wasn’t included in the same clade as the other Stx2d phages (Figure 4). Stx2d-producing *E. coli* have been isolated from ruminants (Tasara et al., 2008) and have also been reported in other animal species (Baranzoni et al., 2016). This may account for a wider ecological niche of this group of Stx phages, also explaining the variability observed in the sequence and gene content of these phages and of their “*stx* regions.”

## Conclusion

In conclusion, we showed that the Stx2 phages analyzed in this study presented a considerable variability and that some of them could have originated from different ancestors that acquired the *stx* genes in multiple transduction events. We make the hypotheses that Stx2a, Stx2c and some Stx2d phages may have derived from the same ancestor and that the “*stx* regions” could present genetic determinants promoting or facilitating the colonization of specific reservoirs and environments by the bacterial host. As it has been proposed by Saile et al. (2018), *nanS-p* could be one of these, and this reinforces the hypothesis of a role for the Stx phages in conferring an advantage to the host bacterial strain. However, we cannot exclude that also other determinants present on the phage’s genome outside the “*stx* region” could have contributed to the adaptation of their bacterial host to specific niches. As a matter of fact, most of the genes present in the different phages studied could not be annotated resulting as hypothetical proteins whose function could not be defined. Therefore, it is certainly possible that the adaptation to the host reservoir could be a multifactorial process implying both genes present on the phage’s genome and the transduced DNA and that *nanS-p* gene could be part of a more complex asset of determinants influencing the fitness of the bacterial host in the reservoir.

While the comparative approach presented here has limitations linked to the scarce availability of genomes of phages encoding some of the Stx subtypes, at the same time it gives indications that may direct further investigations. Further analyses involving an enlarged panel of Stx phage genomes, including those encoding the more recent Stx2 subtypes and the Stx1 phages, would be beneficial for a better comprehension of the mechanisms underlying the evolution of the Stx phages.

## Data Availability Statement

The datasets presented in this study can be found in online repositories. The names of the repository/repositories and accession number(s) can be found at: https://www.ebi.ac.uk/ena, PRJEB37181.

## Author Contributions

MZ performed most of the sequencing and bioinformatic analyses of data and drafted the manuscript. RT contributed to the scientific discussion and particularly in the analysis of the genomic data and to the critical revision of the manuscript. PC prepared and checked the bacterial cultures and performed the Real Time PCR experiments. PQ and AM-V performed the phages’ induction, amplification, and purification. VM contributed to the bioinformatic analyses and to the critical revision of the manuscript. MM supervised all the phages purification activities and actively contributed to the scientific discussion and to the critical revision of the manuscript. SM conceived the study, contributed to the scientific discussion and thoroughly revised the manuscript. All authors contributed to the article and approved the submitted version.

## Conflict of Interest

The authors declare that the research was conducted in the absence of any commercial or financial relationships that could be construed as a potential conflict of interest.

## Footnotes

1 https://www.iss.it/site/aries

2 http://www.bioinformatics.babraham.ac.uk/projects/fastqc/

3 https://cge.cbs.dtu.dk

4 http://tools.bat.infspire.org/circoletto/

5 https://cge.cbs.dtu.dk

## Supplementary Material

FIGURE S1 Illustrates the relationships between the Stx2d and Stx2a phages through the Circoletto software alignment of the respective phage sequences.

TABLE S1 Includes the analysis on the presence or absence of the genes with an annotated function found in all the phages’ genomes investigated.
