# Supplementary figures and images for: Investigation on the Evolution of Shiga Toxin-Converting Phages Based on Whole Genome Sequencing
Source: Front Microbiol. 2020 Jul 10;11:1472. doi: 10.3389/fmicb.2020.01472 (PMC7366253; doi:10.3389/fmicb.2020.01472)

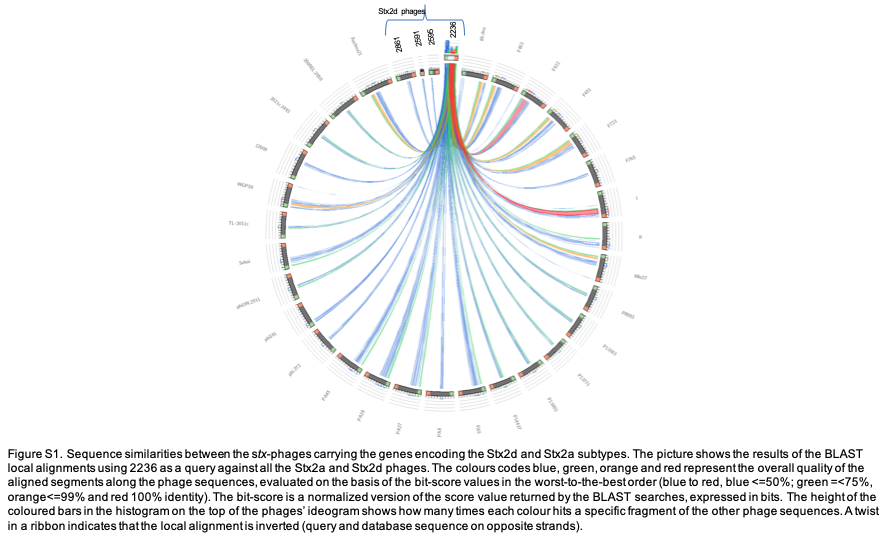

Supplement: FIGURE S1 — Illustrates the relationships between the Stx2d and Stx2a phages through the Circoletto software alignment of the respective phage sequences. [file Image_1.jpg]
